# Comparative and phylogenomic analyses of mitochondrial genomes in Coccinellidae (Coleoptera: Coccinelloidea)

**DOI:** 10.7717/peerj.12169

**Published:** 2021-12-09

**Authors:** Xinghao Li, Nan Song, Heng Zhang

**Affiliations:** College of Plant Protection, Henan Agricultural University, Zhengzhou, Henan, China

**Keywords:** Phylogenetic, Coccinellidae, Mitochondrial genome

## Abstract

The Coccinellidae are one of the most familiar beetle families, the ladybirds. Despite the great ecological and economic significance, the phylogenetic relationships of Coccinellidae remain poorly understood. One of the reasons is that the sequenced mitogenomes available for this family are very limited. We sequenced complete or nearly complete mitogenomes from seven species of the tribe Coccinellini with next-generation sequencing. All species have the same gene content and gene order as the putatively ancestral insect mitogenome. A large intergenic spacer region (> 890 bp) was found located between *trnI* and *trnQ*. The potential for using secondary structures of the large and small ribosomal subunits for phylogenetic reconstruction was predicted. The phylogenetic relationships were explored through comparative analyses across more than 30 coccinellid species. We performed phylogenetic analyses with both concatenation methods (Maximum Likelihood and Bayesian Inference) and multispecies coalescent method (ASTRAL). Phylogenetic results strongly supported the monophyly of Coccinellidae. Within Coccinellidae, the Epilachnini and the Coccinellini including Halyziini were monophyletic, while the Scymnini and Coccidulini were non-monophyletic.

## Introduction

The family Coccinellidae comprises a group of insects often called ladybirds or ladybugs, a familiar family of beetles, for example, the seven-spot ladybird beetle *Coccinella septempunctata* Linnaeus. Currently, the family is subdivided into about 360 genera, with approximately 6,000 species worldwide (Vandenberg, 2002). Many of coccinellid species are beneficial due to their predaceous nature, and are well known for roles in biological control. The predatory group is often referred to as aphidophagous, despite sometimes also feeding on other hemipteran species, for example, scales and whiteflies (Hodek & Honek, 2009; Obrycki et al., 2009). Additionally, other ladybirds are phytophagous, including the genus *Bulaea* Mulsant and the whole subfamily Epilachninae (Giorgi et al., 2009). Some phytophagous ladybird beetles are serious pests and cause damage to important crops, such as *Epilachna varivestris* Mulsant and *Henosepilachna vigintioctopunctata* (Fabricius) (Szawaryn et al., 2015).

Despite the monophyly of the whole Coccinellidae is well supported (Giorgi et al., 2009; Magro et al., 2010; Robertson et al., 2015; Seago et al., 2011; Slipinski, 2007), the phylogenetic relationships within the family are uncertain. Redtenbacher (1844) classified the Coccinellidae into two groups: the phytophagous group and the aphidophagous group. The two groups were further classified into six or seven subfamilies. Sasaji (1968; 1971a; 1971b), based on larval and adult morphological characters, proposed a six subfamilies’ classification system (Sticholotidinae, Coccidulinae, Scymninae, Chilocorinae, Coccinellinae, and Epilachninae). This scheme received widespread acceptance (Giorgi et al., 2009). Kovář (1996) added the Ortaliinae into the scheme of six subfamilies to construct the seven subfamilies scheme. The phytophagous group corresponding to the subfamily Epilachninae is often considered to monophyletic (Giorgi et al., 2009; Seago et al., 2011; Song et al., 2020). The aphidophagous group spans the remaining subfamilies (Giorgi et al., 2009). Within the aphidophagous group, the Coccinellinae is monophyletic, while the other subfamilies are non-monophyletic (Giorgi et al., 2009; Magro et al., 2010).

Previous authors have attempted to reconstruct the phylogenetic relationships among the subfamilies based on morphological (Yu, 1994) and/or molecular (Giorgi et al., 2009; Magro et al., 2010; Robertson et al., 2015; Seago et al., 2011) data. However, most of the subfamilies proposed by Sasaji (1968; 1971a; 1971b) were not supported by recent molecular studies (Giorgi et al., 2009; Magro et al., 2010; Seago et al., 2011; Che et al., 2021). Seago et al. (2011) conducted the simultaneous analysis of morphological and multi-locus molecular data to investigate the phylogenetic relationships among major clades of Coccinellidae. Their results found no statistical support for the classification of several subfamilies, except for Coccinellinae and Microweisinae. Che et al. (2021) recognized three subfamilies in Coccinellidae, namely Microweiseinae, Monocoryninae stat. nov., and Coccinellinae, based on multiple nuclear protein-coding gene sequences. In this study, our taxon sampling was focused on the tribe Coccinellini (24 exemplars out of 40 analyzed coccinellid species). The increased taxon sampling allowed us to investigate the relationships below the subfamily level.

Rapid radiations pose one of the most difficult challenges for the phylogenetic estimation of subfamily relationships (Robertson et al., 2015). For other beetle groups (*e.g.*, Galerucinae and Chrysomelidae) with similar divergence time, some authors have successfully used mitogenomes to resolve the subfamily (Nie et al., 2018), family (Nie et al., 2020) and above the family level relationships (Timmermans et al., 2016). Song et al. (2020) and Yuan et al. (2020) provided the first mitogenome phylogenetic analyses of Coccinellidae. The results confirmed the monophyly of Coccinellinae and Epilachninae. But both studies only considered DNA sequence variation in their phylogenetic reconstructions (Song et al., 2020; Yuan et al., 2020).

The previous research work often used the super matrix to construct the phylogenetic tree of Coccinellidae (*e.g.*, Robertson et al., 2015; Song et al., 2020). Mitochondrial DNA as a phylogenetic marker may suffer from phylogenomic biases as associated with incomplete lineage sorting and gene tree heterogeneity. Multispecies coalescent methods have been developed to tackle these problems. A recent study has applied multispecies coalescent analysis to phylogenetic reconstruction within Lepidoptera based on mitogenome sequence data (Kim, Kim & Cameron, 2020). Their results demonstrated that multispecies coalescent analysis can be a reliable inference method for mitogenomic data in resolving insect phylogenetic relationships. The Coccinellidae have experienced rapid radiation (Robertson et al., 2015; Seago et al., 2011; Tomaszewska et al., 2021). Here, we attempted to use the super tree generated by ASTRAL analysis of mitogenome data to investigate the phylogenetic relationships in Coccinellidae.

Besides applying the alignments of mitochondrial DNA sequences to phylogenetic reconstruction, the mitogenome organization (Shao, Campbell & Barker, 2001), and the secondary structures of mitochondrial large subunit (*rrnL*) and small subunit (*rrnS*) may provide potential information for phylogenetic studies (Buckley et al., 2000; Page, 2000; Page, Cruickshank & Johnson, 2002; Yoshizawa & Johnson, 2003). The use of both *rrnL* and *rrnS* gene sequences in phylogenetic reconstructions is undercut by alignment difficulties. Both rRNA genes contain a number of possibly uninformative conserved regions interspersed with highly compositionally heterogeneous variable regions that are difficult to align. Particularly, it is difficult to align these highly variable regions accurately using the current algorithmic alignment methods (Song et al., 2020). The rRNA molecule forms distinct secondary structures that play an important role in the functioning of ribosomes (Noller, 1984). Secondary structure information can thus be used to identify homologous positions, with higher phylogenetic informativeness.

In this study, we sequenced seven complete or nearly complete mitogenomes of the subfamily Coccinellinae, with three main aims: (1) to compare mitogenome organization and gene content across coccinellid lineages; (2) to identify conserved sequence motifs and the associated secondary structure elements in *rrnL* and *rrnS* genes to provide the potential phylogenetic information; (3) to add more mitogenome data to uncover the evolutionary relationships of ladybirds.

## Materials and Methods

### Taxon sampling

A total of 46 mitogenomes were analyzed in this study: 40 species of ladybird beetles and other six species of Cucujifamia outgroups (Table 1). Of which, seven mitogenomes of the tribe Coccinellini were newly sequenced in this study. We extracted genomic DNA from seven specimens, *Coccinella lama* Kapur, *Hippodamia variegata* (Goeze), *Coccinella transversoguttata* Faldermann, *Adalia bipunctata* (Linnaeus), and *Oenopia dracoguttata* Jing were collected from Lhasa, Tibet, China in June 28, 2019; *Harmonia axyridis* (Pallas) and *Harmonia eucharis* (Mulsant) were collected from Linzhi, Tibet, China in August 3, 2019. Thoracic muscle was used for DNA extraction, using the TIANamp Genomic DNA Kit (TIANGEN BIOTECH CO., LTD), according to the manufacturer’s protocol. Extracted DNA was stored at −20 °C.

**Table 1:** Taxa included in this study.

Item	Family	Subfamily	Tribe	Species	Accession number	Reference
Outgroup	Bothrideridae	Bothriderinae	Bothriderini	*Dastarcus helophoroides*	NC_024271	Zhang et al., 2015
	Corylophidae	Corylophinae	Rypobiini	*Gloeosoma* sp.	JX412843	Unpublished
	Discolomatidae	Discolomatinae	–	Discolomatinae sp.	JX412748	Unpublished
	Endomychidae	Endomychinae	–	*Endomychus coccineus*	JX313667	Unpublished
	Erotylidae	Xenoscelinae		*Loberonotha olivascens*	JX412784	Unpublished
	Latridiidae	Latridiinae	–	*Enicmus brevicornis*	JX313681	Unpublished
Ingroup	Coccinellidae	Scymninae	Scymnini	*Nephaspis* sp. DPP-2018	MG253275	Unpublished
	Coccinellidae	Scymninae	Scymnini	Scymninae sp. 2 ACP-2013	MH940166	Crampton-Platt et al., 2015
	Coccinellidae	Scymninae	Scymnini	*Nephus includens*	MN164642	Magro et al., 2020
	Coccinellidae	Scymninae	Scymnini	*Nephus reunioni*	MN164643	Magro et al., 2020
	Coccinellidae	Scymninae	Scymnini	*Nephus* sp. 1 EL-2020	MN164644	Magro et al., 2020
	Coccinellidae	Scymninae	Scymnini	*Nephus voeltzkowi*	MN164645	Magro et al., 2020
	Coccinellidae	Scymninae	–	Coccinellidae sp. 1 EF-2015	KT780638	Unpublished
	Coccinellidae	Coccidulinae	Coccidulini	*Cryptolaemus montrouzieri*	KT878320	Unpublished
	Coccinellidae	Coccidulinae	Coccidulini	*Coccidula rufa*	JX412767	Unpublished
	Coccinellidae	Ortaliinae	Noviini	*Rodolia quadrimaculata*	MN053055	Song et al., 2019
	Coccinellidae	Epilachninae	Epilachnini	*Afissula* sp. XL-2019	MN053057	Song et al., 2019
	Coccinellidae	Epilachninae	Epilachnini	*Henosepilachna pusillanima*	KJ131489	Behere et al., 2014
	Coccinellidae	Epilachninae	Epilachnini	*Henosepilachna vigintioctopunctata*	MG584727	Unpublished
	Coccinellidae	Epilachninae	Epilachnini	*Subcoccinella vigintiquattuorpunctata*	KT780695	Unpublished
	Coccinellidae	Epilachninae	Epilachnini	*Epilachna admirabilis*	MN053053	Song et al., 2019
	Coccinellidae	Chilocorinae	Chilocorini	*Chilocorus bipustulatus*	MN053054	Song et al., 2019
	Coccinellidae	Coccinellinae	Halyziini	*Halyzia sedecimguttata*	KT780652	Unpublished
	Coccinellidae	Coccinellinae	Halyziini	Halyziini sp. HA	MG584728	Zhang et al., 2017
	Coccinellidae	Coccinellinae	Coccinellini	***Adalia bipunctata***	**MW029465**	**This study**
	Coccinellidae	Coccinellinae	Coccinellini	*Aiolocaria hexaspilota*	MK583344	Seo et al., 2019
	Coccinellidae	Coccinellinae	Coccinellini	*Anatis ocellata*	KX035143	Unpublished
	Coccinellidae	Coccinellinae	Coccinellini	*Anisosticta novemdecimpunctata*	KT876880	Linard et al., 2016
	Coccinellidae	Coccinellinae	Coccinellini	*Calvia championorum*	KX132085	Unpublished
	Coccinellidae	Coccinellinae	Coccinellini	*Calvia decemguttata*	KX087252	Unpublished
	Coccinellidae	Coccinellinae	Coccinellini	*Cheilomenes sexmaculata*	KM244706	Tang et al., 2014
	Coccinellidae	Coccinellinae	Coccinellini	*Coccinella septempunctata*	JQ321839	Kim et al., 2011
	Coccinellidae	Coccinellinae	Coccinellini	***Coccinella lama***	**MW029464**	**This study**
	Coccinellidae	Coccinellinae	Coccinellini	***Coccinella transversoguttata***	**MW029466**	**This study**
	Coccinellidae	Coccinellinae	Coccinellini	*Coelophora saucia*	MN053056	Song et al., 2019
	Coccinellidae	Coccinellinae	Coccinellini	*Cycloneda sanguinea*	KU877170	Unpublished
	Coccinellidae	Coccinellinae	Coccinellini	*Eriopis connexa*	MG253268	Unpublished
	Coccinellidae	Coccinellinae	Coccinellini	*Harmonia quadripunctata*	KX087296	Unpublished
	Coccinellidae	Coccinellinae	Coccinellini	***Harmonia axyridis***	**MW029463**	This study
	Coccinellidae	Coccinellinae	Coccinellini	***Harmonia eucharis***	**MW029462**	This study
	Coccinellidae	Coccinellinae	Coccinellini	*Hippodamia convergens*	KX755332	Unpublished
	Coccinellidae	Coccinellinae	Coccinellini	*Hippodamia undecimnotata*	KX087298	Unpublished
	Coccinellidae	Coccinellinae	Coccinellini	***Hippodamia variegata***	**MW029468**	**This study**
	Coccinellidae	Coccinellinae	Coccinellini	***Oenopia dracoguttata***	**MW029467**	**This study**
	Coccinellidae	Coccinellinae	Coccinellini	*Propylea japonica*	KM244660	Tang et al., 2014
	Coccinellidae	Coccinellinae	Coccinellini	*Propylea* sp. HSL-2016	KX132084	Unpublished

**Notes.**

Bold denotes the newly sequenced species.

### Library construction and next-generation sequencing

About 500 ng genomic DNA of the individual species were used for library preparation using the Illumina TruSeq TM DNA Sample Prep Kit (Illumina, San Diego, CA, USA), according to manufacturer’s instructions. DNA were sheared into 350-bp fragments in a Covaris M220 instrument (Covaris Inc.). Libraries were sequenced on the Illumina HiSeq 2500 platform with 150-bp paired-end reads, at Shanghai OE Biotech. Co., Ltd, China.

### Mitogenome assembly and annotation

Statistics for next-generation sequencing are presented in Table 2. Adapters and low-quality reads were trimmed from raw data by using NGS Toolkit (Patel & Jain, 2012). High-quality reads (Q20 ≥ 97.71%, and Q30 ≥ 93.43%) were used in subsequent genome assemblies in Geneious R11, with the following parameters: iterate up to 100 times (slow), maximum gaps per read 5%, maximum gap size 20 bp, minimum overlap 50 bp, and minimum overlap identity 95%. The mtDNA sequence of *Cycloneda sanguinea* (Linnaeus) (accession number: KU877170) was used as reference for assembly.

Preliminary mitogenome annotations were conducted in MITOS web (Bernt et al., 2013), under the default settings and the invertebrate genetic code for mitochondria. The gene boundaries of protein-coding and ribosomal RNA were refined by alignment against published Coccinellini mitogenome sequences. Transfer RNAs (tRNA) were annotated in MITOS web (Bernt et al., 2013), with secondary structures inferred. Secondary structures for *rrnL* and *rrnS* genes were predicted by reference to the darkling beetle *Gonocephalum outreyi* Chatanay (Song et al., 2018), and figured manually in Adobe Illustrator CS6. Annotated mitogenome sequences have been submitted to GenBank with the accession numbers of MW029462–MW029468.

**Table 2:** Statistics of next-generation sequencing.

Species name	Total number of sequenced raw paired reads	Raw Q30	Total number of clean paired reads	Clean Q30
*Coccinella lama*	18.52 Mbp	84.02%	15.57 Mbp	90.41%
*Hippodamia variegata*	20.07 Mbp	85.28%	17.14 Mbp	91.12%
*Coccinella transversoguttata*	21.61 Mbp	85.15%	18.45 Mbp	91.00%
*Adalia bipunctata*	19.47 Mbp	84.58%	16.53 Mbp	90.72%
*Oenopia dracoguttata*	22.48 Mbp	87.47%	19.24 Mbp	91.98%
*Harmonia axyridis*	17.24 Mbp	83.73%	14.47 Mbp	90.28%
*Harmonia eucharis*	21.28 Mbp	86.22%	18.57 Mbp	91.52%

### Phylogenetic analyses

Before sequence alignment, we added the mitochondrial genes of the seven newly sequenced coccinellid species into the dataset constructed from the mitognome sequences downloaded from GenBank. For the phylogenetic analyses, we concatenated nucleotide alignments from all 37 mitochondrial genes. Protein-coding genes were individually aligned using MAFFT (Katoh & Standley, 2013) in the TranslatorX (Abascal, Zardoya & Telford, 2010) server. Each tRNA, and rRNA was aligned in the MAFFT (E-INS-I algorithm) alignment server and adjusted following reference to secondary structural models. Ambiguously aligned sites in each alignment were removed with Gblocks (Talavera & Castresana, 2007), using the less stringent selection option. Alignments were concatenated using FASconCAT_v1.0 (Kueck & Meusemann, 2010). The sequence alignment used in the phylogenetic analyses is provided in the Supplementary File 1.

Phylogenetic relationships were inferred using Maximum Likelihood (ML) and Bayesian Inference (BI). PartitionFinder 2 (Lanfear et al., 2017) was used to select best-fitting partition schemes and corresponding substitution models for the concatenated alignment (Table S1). Data blocks were defined by codon position and by gene for protein-coding genes. All 22 tRNA genes were included in a single partition, while each of rRNA genes was defined as a separate partition. PartitionFinder analysis was conducted with the CIPRES web portal (Miller, Pfeiffer & Schwartz, 2010), using the corrected Akaike information criterion (AICc). ML analysis was performed using IQ-TREE (Lam-Tung et al., 2015; Nguyen et al., 2015; Trifinopoulos et al., 2016) at the CIPRES web portal (Miller, Pfeiffer & Schwartz, 2010). Nodal supports were estimated with 10,000 ultrafast bootstrap replicates (Hoang et al., 2018). The SH-aLRT branch test (Guindon et al., 2010) was conducted with 1,000 replicates. The command -spp was employed to consider the FreeRate heterogeneity model.

BI analysis was performed with PhyloBayes MPI (Lartillot et al., 2013) implemented on the CIPRES web portal. The site-heterogeneous CAT-GTR model (Lartillot & Philippe, 2004) was employed, with constant sites removed. Two independent Markov chain Monte Carlo (MCMC) chains starting from a random tree were run for 20,000 generations. The initial 20% cycles in each MCMC chain were discarded as burn-in. A consensus tree was computed from the remaining trees. Convergence of the two chains was indicated by a “maxdiff” value of 0.1.

We used ASTRAL v 5.7.1 (Mirarab et al., 2014; Zhang et al., 2018) to estimate a species tree. ML tree searches were conducted for individual gene alignments (13 protein-coding genes, two rRNA genes as single genes plus the 22 tRNA genes combined as a single alignment), with IQ-TREE. Gene trees were then used as input for ASTRAL, using bootstrap replicates from the IQ-TREE estimated gene trees for branch support values estimation.

## Results

### Genome sequencing and mitogenome assembling

For the newly sequenced species, the total number of raw reads varied between 17.24 Mbp (*H. axyridis*) and 22.48 Mbp (*O. dracoguttata*) (Table 2). The proportion of raw reads with phred scores equal to or greater than Q30 ranged between 83.73–87.47%. After filtering low-quality data, the total number of clean reads ranged from 14.47 Mbp to 19.24 Mbp. The proportion of cleaned reads with phred scores equal to or greater than Q30 ranged between 90.28–91.98%.

Of the clean reads, 0.75% (*O. dracoguttata*) to 2.19% (*C. magnifica*) corresponded to mitochondrial reads. Length of assembled mitogenomes varied between 17,963 bp (*C. transversoguttata*) and 21,391 bp (*H. eucharis*) (Table 3). The average values for sequencing depth of mitogenomes ranged from 501-fold (*H. eucharis*) to 1,251-fold (*C. magnifica*). Sequencing depth was not closely correlated with the mitogenome length. The current sequencing depth was sufficient to cover the entire mitogenome.

### General characteristics of mitogenome

All seven newly-sequenced mitogenomes contained the typical 37 mitochondrial genes and a complete control region (Fig. S1). All seven coccinelid mitogenomes had the same arrangement of protein-coding genes, tRNA genes, and rRNA genes as the putatively ancestral insect mitogenome (Cameron, 2014a; Cameron, 2014b).

Nucleotide composition was strongly biased toward A and T. The average A+T content for the whole mitogenomes was 77.9%, made up of 76.5% in the protein-coding genes, 78.9% in the tRNA genes, 78.1% in the rRNA genes and 82.5% in the control region. Twelve of the 13 protein-coding genes started with the typical codon ATN (ATT, ATG, and ATA). However, for *cox1*, the putative initiation codon was TCG(Ser) in all newly sequenced mitogenomes. Canonical stop codons (TAG and TAA) were present for 11/12 protein-coding genes depending on species. Incomplete stop codons (T or TA) were inferred for *cox2* (*H. variegata* and *H. eucharis*), *cox3* (*C. lama* and *C. transversoguttata*), *nad1* (*O. dracoguttata* and *H. eucharis*), and *nad2* (*H. axyridis*).

**Figure 1:**
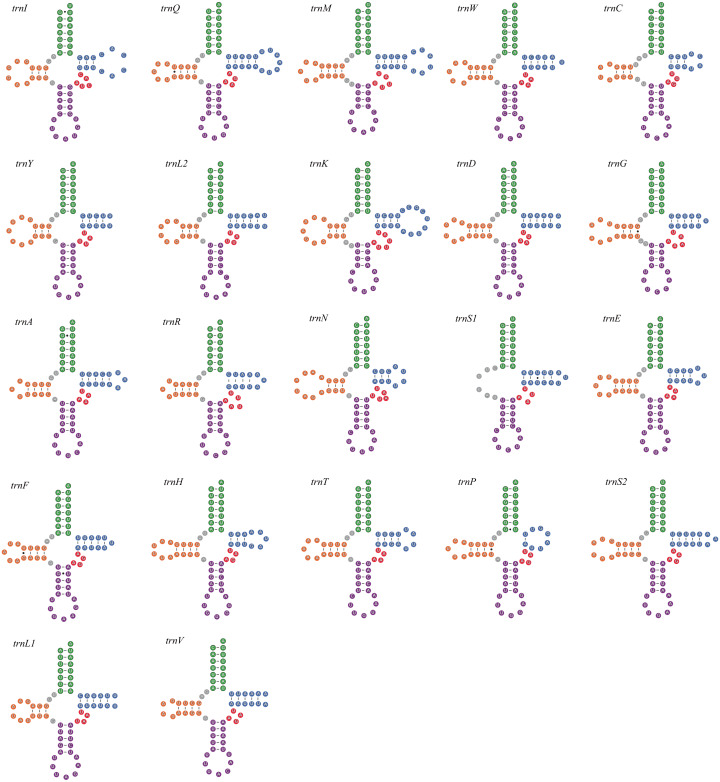
The secondary structures of tRNA genes inferred for *Coccinella lama*.

All seven newly-sequenced mitogenomes had a full set of 22 mitochondrial tRNA genes, which ranged from 50 bp (*trnH*, *A. bipunctata*) to 71 bp (*trnK*, *A. bipunctata*, *H. axyridis* and *H. eucharis*) in size. All of mitochondrial tRNA genes had secondary structures commonly seen in other insects, with the exception for *trnS1*, which lacked a complete dihydrouridine (DHU) arm (*e.g.*, *C. lama* in Fig. 1).

**Table 3:** Assembling results, statistics of mapping, and the proportion of the total sequencing reads mapped to the mitogenomes.

Specis	Mitogenome (bp)	Mapped reads	Minimum sequencing depth	Average sequencing depth	Mitochondrial reads (%)
*Coccinella lama*	19,337	170,237	169	1,251	2.19
*Hippodamia variegata*	18,347	127,665	136	993	1.49
*Coccinella transversoguttata*	17,963	79,131	121	642	0.86
*Adalia bipunctata*	18,750	117,955	167	941	1.43
*Oenopia dracoguttata*	19,359	72,567	55	550	0.75
*Harmonia axyridis*	18,737	93,440	549	729	1.29
*Harmonia eucharis*	21,391	71,886	68	501	0.77

**Figure 2:**
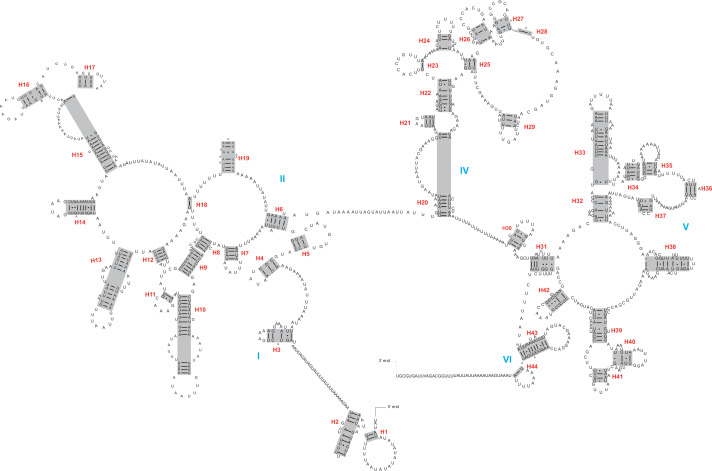
The secondary structure of *rrnL* inferred for *Harmonia axyridis*. Red numbers denote the helices and blue Roman numerals denote the domains.

**Figure 3:**
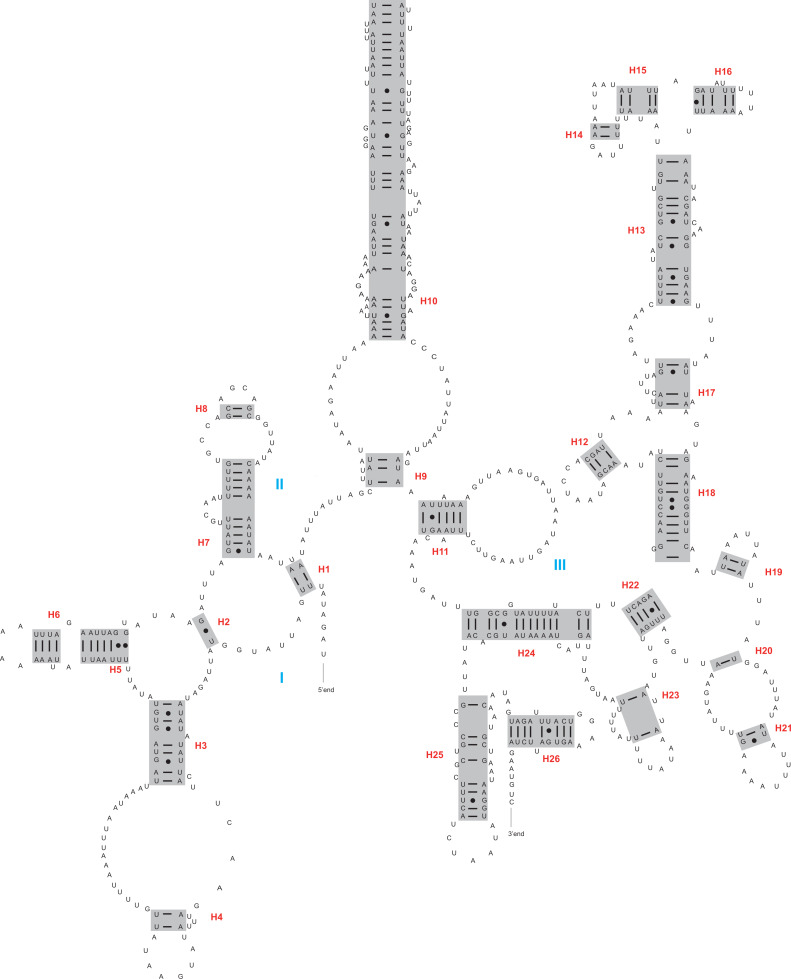
The secondary structure of *rrnS* inferred for *Harmonia axyridis*. Red numbers denote the helices and blue Roman numerals denote the domains.

The *rrnL* and *rrnS* genes were 1,200–1,299 bp and 746–762 bp, respectively. The secondary structures inferred for the *rrnL* and *rrnS* genes were similar to the secondary structure models proposed for other beetles (*e.g.*, *G. outreyi*, Song et al., 2018) (Figs. 2–3 and Fig. S2). In the *rrnL* genes, there were differences in the number of helices. *H. variegate*, *A. bipunctata*, *O. dracoguttata* and *H. axyridis* had 44 helices. *C. lama* and *C. transversoguttata* had 45 helices, while *H. eucharis* had 43 helices. Base mismatches and sequence length variation resulted in observed differences. Each *rrnL* gene contained five domains (I-II, IV-VI), and lacked domain III. *rrnS* genes had three domains (I, II, III) composed of 26 helices.

The complete control region between *rrnS* and *trnI* was identified within assembled mitogenomes, demonstrating circulation of the molecule. Besides the control region, a large non-coding region (or intergenic spacer region) was present between *trnI* and *trnQ*. The sequence lengths of this region varied between 895 bp (*C. transversoguttata*) and 2,745 bp (*C. lama*).

### Mining phylogenetic information from mitochondrial ribosomal RNA secondary structures

**Figure 4:**
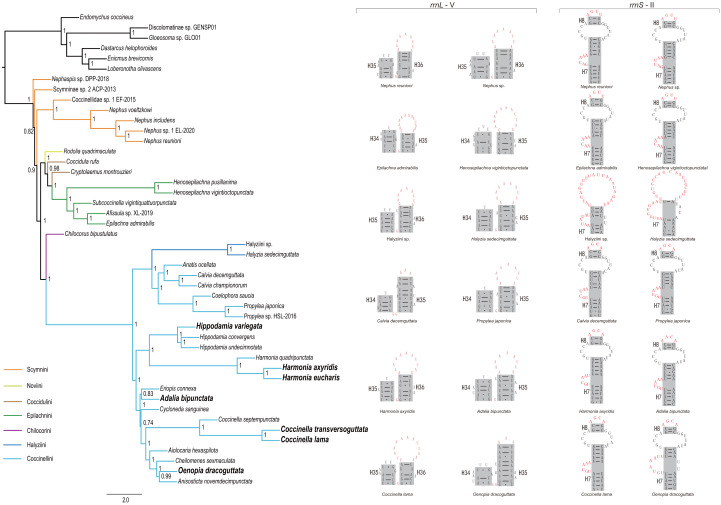
Phylogenetic relationships within Coccinellidae inferred using ASTRAL analysis (left), and comparisons of the secondary structures of *rrnL* (middle) and *rrnS* (right). In the phylogenetic tree, numbers around nodes indicate the local posterior probabilities. Bold denotes the newly sequenced species.

Through comparison of 34 mitochondrial *rrnL* and 31 *rrnS* secondary structures of coccinellid species, we found that species in the same genus shared conserved motifs. For *rrnL* domain V, the loop located in the tip of helix 35 had largely identical nucleotide composition within a single genus (Fig. 4 middle and Fig. S3), but it was distinguisable between genera. Within Coccinellinae, two species from the tribe Halyziini shared the secondary structure character of *rrnS* helix 7 (Fig. 3). Additionally, they did not have the *rrnS* helix 8, that was distinct from all other Coccinellidae. Although the structures of *rrnS* helix 8 of Coccinellini are basically similar to Epilachnini/Scymnini, there is a discrepancy of two nucleotides (AGUU *vs* AGCA) between them (Fig. 4). In addition to the secondary structures illustrated in Fig. 4, we found that *rrnS* helix 4 also contained potential phylogenetic information (Fig. S4). The Coccinellini shared an identical nucleotide composition of helix 4, while it was distinguishable from other lineages.

### Phylogenetic inference

**Figure 5:**
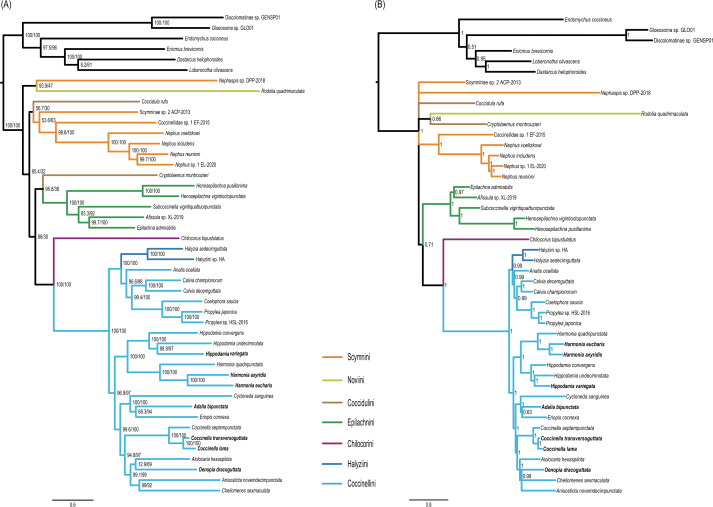
Phylogenetic trees inferred from the super matrix constructed by 37 mitochondrial genes. (A) Maximum Likelihood analysis was performed using IQ-tree. Numbers around nodes indicate support values from SH-aLRT test (left) and ultrafast bootstrap analysis. (B) Bayesian Inference was performed using PhyloBayes. Numbers around nodes indicate posterior probabilities.

Monophyly of the Coccinellidae was strongly supported in both ML and BI analyses (BS = 100, PP = 1, Fig. 5). Within Coccinellidae, the Chilocorini was recovered as the sister group to a clade comprising Halyziini and Coccinellini. This large clade was sister to Epilachnini. However, the sister-group relationship received no statistical support (BS = 30, PP = 0.71). The Epilachnini was consistently supported as monophyletic (BS = 100, PP = 1). In the ML analysis, the *Cryptolaemus* was sister to Epilachnini (BS = 38). A clade comprising a part of Scymnini (*Nephaspis*) and the species representing Noviini (*Rodolia quadrimaculata*) emerged as sister to the rest of the family. But these relationships received no statistical supports (BS = 47). Similarly, in the BI analysis, the basal relationships within Coccinellidae were ambiguous. In particular, relationships among Scymnini, Noviini and Coccidulini were unresolved. The Scymnini and Coccidulini were non-monophyletic.

The Coccinellini including Halyziini formed a large clade. In both ML and BI analyses, the Halyziini was sister to a clade comprising *Anatis*, *Calvia*, *Coelophora* and *Propylea* (BS = 100, PP = 0.99). All genera with more than two species included in this study (*i.e., Halyzia*, *Calvia*, *Propylea*, *Harmonia*, *Hippodamia* and *Coccinella*) were supported as monophyletic (BS = 100, PP =1).

ASTRAL analysis showed better performance in resolving deeper nodes in the Coccinellidae (Fig. 4) with respect to nodal support. Although the Scymnini was still non-monophyletic, the majority of this tribe formed a paraphyletic grade relative to the remaining coccinellid lineages. The two exemplars of Coccidulini formed a paraphyletic grade to Epilachnini. In comparison, members of the Scymnini and Coccidulini were scattered on the trees from both ML and BI analyses. Other difference between multispecies coalescent analysis and the concatenated analyses under ML and BI criteria was the nodal support. The tree from multispecies coalescent analysis typically had higher nodal support values for the internal nodes.

## Discussion

### Large intergenic spacer region in mitogenome

Large intergenic spacer regions have been frequently found in insect mitogenomes (Du et al., 2017; Song et al., 2020; Wang et al., 2019; Wei et al., 2010). In the present study, all newly sequenced coccinellid species had a large intergenic spacer located between *trnI* and *trnQ*. The position of the intergenic spacers accorded with our prior study (Song et al., 2020). Longer sequence lengths in this region contribute to the larger size of the whole mitogenomes of the species in this study. A 54 bp spacer region between *trnQ* and *nad2* was found in the mitogenome of *Manduca sexta* (Linnaeus) (Lepidoptera: Sphingidae) in a previous study (Cameron & Whiting, 2008). The position of this spacer region is adjacent to the large intergenic spacer region found in coccinellid species sequenced in this study. The phylogenetic utility of this arrangement can be evaluated by sequencing and comparing more mitogenomes from related insect groups in future studies.

### Phylogenetic inference

In this study, traditional concatenation methods of analyzing mitogenomes failed to resolve the relationships between the Scymnini and Coccidulini in the BI tree. This result is consistent with other work that found no support for the delimitation of most subfamilies in Coccinellidae (Giorgi et al., 2009; Magro et al., 2010; Seago et al., 2011). Due to mitogenome data availability, the taxon sampling of Scymnini and Coccidulini is still limited. In future studies, we need to sequence more species from both groups to confirm the result. Here we expanded sampling within the Coccinellini, which continued to robustly support the monophyly of the subfamily Coccinellinae and of Coccinellinae genera. This demonstrates that mitogenomic data can be effective in resolving relationships below the subfamily level within the Coccinellidae.

Recent studies recovered members of the former tribe Halyziini within Coccinellini (Nattier et al., 2021; Tomaszewska et al., 2021). Some authors have proposed that Coccinellini, including Halyziini, constitutes a monophyletic group (Che et al., 2021; Nattier et al., 2021; Seago et al., 2011; Tomaszewska et al., 2021). The present study recovered a similar branching pattern based on the mitogenome data, with Halyziini and Coccinellini being placed in a clade. This result is congruent with the results of phylogenetic reconstruction based on combining analyses of nuclear and mitochondrial gene fragments (Nattier et al., 2021; Tomaszewska et al., 2021).

Giorgi et al. (2009) recovered the monophyly of Coccinellinae, based on the *18S* and *28S* rRNA sequences. Magro et al. (2010), based on multiple gene sequence data (nuclear *18S*, *28S* rRNA, and mitochondrial *rrnS*, *rrnL* and *cox1*), also supported the Coccinellinae as a monophyletic group. Our result is consistent with the previous studies. The current mitogenome data supported Epilachnini as monophyletic. This was congruent with Che et al. (2021), but contrasted with Magro et al. (2010). The Epilachnini was paraphyletic in the analyses of Magro et al. (2010). The Scymnini was non-monophyletic across our analyses. This arrangement was also retrieved in the previous studies based on nuclear gene (Che et al., 2021; Giorgi et al., 2009; Robertson, Whiting & McHugh, 2008) and combined data of nuclear and mitochondrial gene sequences (Magro et al., 2010). A sister group relationship between the *Chilocorus* (Chilocorini) and the clade Coccinellini + Halyziini was supported by the present mitogenome analyses. This arrangement is congruent with Escalona et al. (2017), Magro et al. (2010) and Seago et al. (2011), but contrasted with the morphological analyses of Sasaji (1968; 1971a; 1971b).

Multispecies coalescent analysis with ASTRAL showed improved performance in recovering relationships within Coccinellidae, especially for the basal relationships among the lineages of Scymnini and Coccidulini. In addition, the sister group relationship between Chilocorini and Coccinellini was robustly supported. Within Coccinellini, the sister-group relationships between *Coelophora* and *Propylea*, between *Hippodamia* and *Harmonia* were supported, consistent with the prior studies by Escalona et al. (2017), Nattier et al. (2021) and Tomaszewska et al. (2021).

### Implications of mitochondrial rRNA secondary structures for phylogenetic relationships

Exploration of meaningful characters for phylogenetic analysis can be essential to better understand insect evolution. As illustrated for this the secondary structures inferred for this study, two classes of regions are observed in rRNA molecules: the double-stranded stems and single-stranded loops. It has been long debated whether stem characters or loop characters contain useful information for phylogenetic inference (Dixon & Hillis, 1993; Smith, 1989; Wheeler & Honeycutt, 1988). Our analyses showed that loop characters of mitochondrial rRNA genes are phylogenetically informative for the Coccinellidae.

## Conclusions

In the present study, we utilized NGS data to reconstruct complete or nearly complete mitogenome of the Coccinellidae. The mitogenomes are very large, ranging from 17,963 bp (*C. transversoguttata*) to 21,391 bp (*H. eucharis*), due to a large intergenic spacer (>890 bp) located between *trnI* and *trnQ*. The mitochondrial rRNA secondary structures were compared to provide a better phylogenetic alignment. Besides the control region between *rrnS* and *trnI* often found in insect mitogenomes, the presence of a large intergenic region in other position is interesting and further studies are needed to investigate the underlying mechanisms creating and preserving such mitochondrial arrangements. The newly sequenced mitogenomes also contribute to a better understanding of the phylogenetic relationships and evolutionary history of ladybirds. The monophyly of Coccinellidae and of several genera within it are recovered, the bootstrap support value has reached 100. Despite this, increased taxon sampling from other species of the coccinellid group other than Coccinellini is needed to comprehensively evaluate the phylogenetic relationships in Coccinellidae.

## Supplemental Information

10.7717/peerj.12169/supp-1 Supplemental Information 1 Organizational maps of the seven new mitogenomes sequenced in this study

10.7717/peerj.12169/supp-2 Supplemental Information 2 The secondary structure of *rrnL* inferred for *Coccinella magnifica* Red numbers denote the helices and blue roman numbers denote the domains.

10.7717/peerj.12169/supp-3 Supplemental Information 3 The secondary structure of *rrnS* inferred for *Coccinella magnifica* Red numbers denote the helices and blue roman numbers denote the domains.

10.7717/peerj.12169/supp-4 Supplemental Information 4 The secondary structure of *rrnL* inferred for *Hippodamia variegate* Red numbers denote the helices and blue roman numbers denote the domains.

10.7717/peerj.12169/supp-5 Supplemental Information 5 The secondary structure of *rrnS* inferred for *Hippodamia variegate* Red numbers denote the helices and blue roman numbers denote the domains.

10.7717/peerj.12169/supp-6 Supplemental Information 6 The secondary structure of *rrnL* inferred for *Coccinella transversoguttata* Red numbers denote the helices and blue roman numbers denote the domains.

10.7717/peerj.12169/supp-7 Supplemental Information 7 The secondary structure of *rrnS* inferred for *Coccinella transversoguttata* Red numbers denote the helices and blue roman numbers denote the domains.

10.7717/peerj.12169/supp-8 Supplemental Information 8 The secondary structure of *rrnL* inferred for *Adalia bipunctata* Red numbers denote the helices and blue roman numbers denote the domains.

10.7717/peerj.12169/supp-9 Supplemental Information 9 The secondary structure of *rrnS* inferred for *Adalia bipunctata* Red numbers denote the helices and blue roman numbers denote the domains.

10.7717/peerj.12169/supp-10 Supplemental Information 10 The secondary structure of *rrnL* inferred for *Oenopia dracoguttata* Red numbers denote the helices and blue roman numbers denote the domains.

10.7717/peerj.12169/supp-11 Supplemental Information 11 The secondary structure of *rrnS* inferred for *Oenopia dracoguttata* Red numbers denote the helices and blue roman numbers denote the domains.

10.7717/peerj.12169/supp-12 Supplemental Information 12 The secondary structure of *rrnL* inferred for *Harmonia eucharis* Red numbers denote the helices and blue roman numbers denote the domains.

10.7717/peerj.12169/supp-13 Supplemental Information 13 The secondary structure of *rrnS* inferred for *Harmonia eucharis* Red numbers denote the helices and blue roman numbers denote the domains.

10.7717/peerj.12169/supp-14 Supplemental Information 14 Alignments of sequences of *rrnL* helix 35

10.7717/peerj.12169/supp-15 Supplemental Information 15 Comparative analysis of the secondary structures of helix 4 in the *rrnS*

10.7717/peerj.12169/supp-16 Supplemental Information 16 Comparative analysis of the secondary structures of helix 4 in the *rrnS*

10.7717/peerj.12169/supp-17 Supplemental Information 17 The partitioning schemes and best-fitting modes selected by PartitionFinder for the nucleotide dataset PCGRNA

10.7717/peerj.12169/supp-18 Supplemental Information 18 Sequence Data

10.7717/peerj.12169/supp-19 Supplemental Information 19 The new mitogenome sequences determined in this study

## Additional Information and Declarations

### DNA Deposition

The following information was supplied regarding the deposition of DNA sequences: The mtDNA sequence of Coccinellidae are available at GenBank: MW029462 to MW029468.

### Data Availability

The following information was supplied regarding data availability: The raw measurements are available in the Supplementary File.
