# Supplementary material for: Comparative and phylogenomic analyses of mitochondrial genomes in Coccinellidae (Coleoptera: Coccinelloidea)
Source: PeerJ. 2021 Dec 9;9:e12169. doi: 10.7717/peerj.12169 (PMC8667754; doi:10.7717/peerj.12169)
Supplement: Supplemental Information 1 [file peerj-09-12169-s001.pdf]

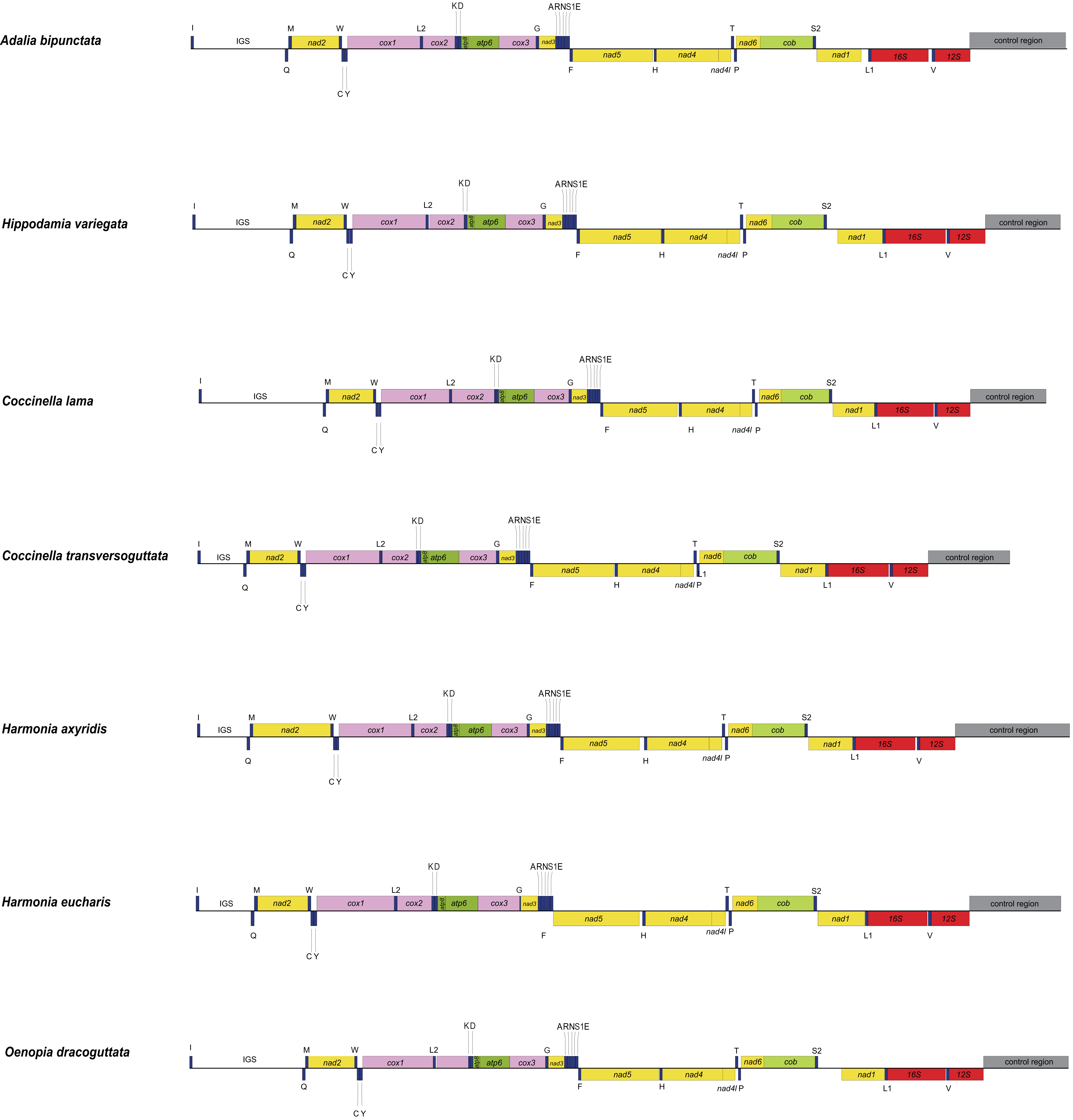

Adalia bipunctata
I IGS M nad2 W cox1 L2 cox2 KD atp8 atp6 cox3 G nad3 ARNS1E T nad6 cob S2 control region
Q CY F nad5 H nad4 nad4l P nad1 L1 16S V 12S
Hippodamia variegata
I IGS M nad2 W cox1 L2 cox2 KD atp8 atp6 cox3 G nad3 ARNS1E T nad6 cob S2 control region
Q CY F nad5 H nad4 nad4l P nad1 L1 16S V 12S
Coccinella lama
I IGS M nad2 W cox1 L2 cox2 KD atp8 atp6 cox3 G nad3 ARNS1E T nad6 cob S2 control region
Q CY F nad5 H nad4 nad4l P nad1 L1 16S V 12S
Coccinella transversoguttata
I IGS M nad2 W cox1 L2 cox2 KD atp8 atp6 cox3 G nad3 ARNS1E T nad6 cob S2 control region
Q CY F nad5 H nad4 nad4l P L1 nad1 L1 16S V 12S
Harmonia axyridis
I IGS M nad2 W cox1 L2 cox2 KD atp8 atp6 cox3 G nad3 ARNS1E T nad6 cob S2 control region
Q CY F nad5 H nad4 nad4l P nad1 L1 16S V 12S
Harmonia eucharis
I IGS M nad2 W cox1 L2 cox2 KD atp8 atp6 cox3 G nad3 ARNS1E T nad6 cob S2 control region
Q CY F nad5 H nad4 nad4l P nad1 L1 16S V 12S
Oenopia dracoguttata
I IGS M nad2 W cox1 L2 KD atp8 atp6 cox3 G nad3 ARNS1E T nad6 cob S2 control region
Q CY F nad5 H nad4 nad4l P nad1 L1 16S V 12S
